# Increased expression of S100A6 at the invading fronts of the primary lesion and liver metastasis in patients witholorectal adenocarcinoma

**DOI:** 10.1054/bjoc.2000.1356

**Published:** 2000-08-17

**Authors:** K Komatsu, Y Kobune-Fujiwara, A Andoh, S Ishiguro, H Hunai, N Suzuki, M Kameyama, K Murata, J Miyoshi, H Akedo, M Tatsuta, H Nakamura

**Affiliations:** Departments of Tumor Biochemistry, Molecular Biology, Research Institute; Departments of Pathology, Surgery, Gastrointestinal Oncology, Osaka Medical Center for Cancer and Cardiovascular Diseases, 1–3–3 Nakamichi, Higashinari-ku, Osaka, 537–8511, Japan

**Keywords:** S100A6 expression, colorectal adenocarcinoma, invading front

## Abstract

Two members of the S100 gene family, S100A6 and S100A4 have been suggested to be associated with cancer invasion and metastasis. To study their involvement in the malignancy of human colorectal adenocarcinoma, we examined the protein expression levels of S100A6 and S100A4 in the primary colorectal adenocarcinoma (T) and paired adjacent normal colorectal mucosa (N) from 12 cases, quantitatively by Western blot analysis. In 11 of 12 and seven of 12 cases, S100A6 and S100A4 expression levels were higher in T than in N, respectively. Average S100A6 level in T was significantly higher than in N (about × 2.3;*P*= 0.001), whereas average S100A4 level in T was not. When S100A6 expression levels in three sets of matched samples of primary colorectal adenocarcinoma (T) and liver metastasis (M) were examined, S100A6 levels were higher in M than in T in two of three cases. Immunohistochemical analysis using monoclonal anti-S100A6 antibody showed that 23 of 42 (55%) primary colorectal adenocarcinoma and 15 of 16 (94%) liver metastasis specimens were positively stained. S100A6 immunostaining of primary colorectal adenocarcinomas was significantly more intense in the invading fronts with structural atypia than in central portions with glandular structure (*P*< 0.0001), whereas Ki-67 staining (a growth marker) was similar in these two portions. Interestingly, S100A6 and Ki-67 immunostaining patterns in liver metastases were also the same as in primary lesions. These results suggest that S100A6 is involved in the invasive process of human colorectal adenocarcinomas and that S100A6 expression levels decrease when carcinoma cells form glandular structure again at the central portions of metastatic nodules. © 2000 Cancer Research Campaign


*British Journal of Cancer* (2000) **83**(6), 769–774
© 2000 Cancer Research Campaign
doi: 10.1054/ bjoc.2000.1356, available online at http://www.idealibrary.com on IDEAL®

### Increased expression of S100A6 at the invading fronts of the primary lesion and liver metastasis in patients with colorectal adenocarcinoma

**K Komatsu[1], Y Kobune-Fujiwara[1], A Andoh[1], S Ishiguro[2], H Hunai[2], N Suzuki[2], M Kameyama[3], K Murata[3], J Miyoshi[4], H Akedo[1], M Tatsuta[5] and H Nakamura[1]**

Departments of [1]Tumor Biochemistry and [4]Molecular Biology, Research Institute; Departments of [2]Pathology, [3]Surgery and [5]Gastrointestinal Oncology, Osaka Medical Center for Cancer and Cardiovascular Diseases, 1–3–3 Nakamichi, Higashinari-ku, Osaka 537–8511, Japan

**Summary** Two members of the S100 gene family, S100A6 and S100A4 have been suggested to be associated with cancer invasion and metastasis. To study their involvement in the malignancy of human colorectal adenocarcinoma, we examined the protein expression levels of S100A6 and S100A4 in the primary colorectal adenocarcinoma (T) and paired adjacent normal colorectal mucosa (N) from 12 cases, quantitatively by Western blot analysis. In 11 of 12 and seven of 12 cases, S100A6 and S100A4 expression levels were higher in T than in N, respectively. Average S100A6 level in T was significantly higher than in N (about $\times$ 2.3; $P = 0.001$), whereas average S100A4 level in T was not. When S100A6 expression levels in three sets of matched samples of primary colorectal adenocarcinoma (T) and liver metastasis (M) were examined, S100A6 levels were higher in M than in T in two of three cases. Immunohistochemical analysis using monoclonal anti-S100A6 antibody showed that 23 of 42 (55%) primary colorectal adenocarcinoma and 15 of 16 (94%) liver metastasis specimens were positively stained. S100A6 immunostaining of primary colorectal adenocarcinomas was significantly more intense in the invading fronts with structural atypia than in central portions with glandular structure ($P < 0.0001$), whereas Ki-67 staining (a growth marker) was similar in these two portions. Interestingly, S100A6 and Ki-67 immunostaining patterns in liver metastases were also the same as in primary lesions. These results suggest that S100A6 is involved in the invasive process of human colorectal adenocarcinomas and that S100A6 expression levels decrease when carcinoma cells form glandular structure again at the central portions of metastatic nodules. © 2000 Cancer Research Campaign

**Keywords**: S100A6 expression; colorectal adenocarcinoma; invading front

S100 proteins are low molecular weight $Ca^{2+}$-binding proteins. They are thought to activate target proteins and therefore influence cellular response along the calcium signal transduction pathway (Zimmer et al, 1995; Shäfer and Heizmann, 1996). Among these S100 proteins, S100A6 (also known as calcyclin/2A9/5B10/PRA) and S100A4 (also known as pEL98/mtsl/p9Ka/18A2/42A/calvasculin/FSP1/CAPL) have been reported to be involved in invasion and metastasis of cancer cells.

S100A6 was initially identified as mRNA preferentially expressed in proliferating rather than quiescent cells (Calabretta et al, 1986; Jackson-Grusby et al, 1987). It has been shown that increased S100A6 expression is associated with metastatic phenotype of NIH-3T3 cells (Guo et al, 1990) and the metastatic behaviour of human melanoma cells (Weterman et al, 1992; 1993).

S100A4 has been isolated by subtractive hybridization of non-metastatic cells from metastatic mouse mammary adenocarcinoma cells (Ebralidze et al, 1989). Rat epithelial cells transfected with S100A4 cDNA showed metastatic capacity in syngeneic rats upon injection in the mammary fat pad (Davies et al, 1993). Strong evidence supporting a role for S100A4 in metastasis was obtained in experiment where ribozyme-induced inhibition of S100A4 expression reversed the in vivo metastatic phenotype of human osteosarcoma cells (Maelandsmo et al, 1996).

By immunohistochemical analysis, we have previously shown that S100A6 expression in human colorectal adenocarcinomas is significantly correlated with two clinical parameters, lymphatic permeation and Duke's tumour status (Komatsu et al, 2000). Although there have been several reports on S100A6 and S100A4 expression in human cancer tissues, their expression levels have been measured semiquantitatively by Northern blot analysis or by immunohistochemistry. In the present study, we examined their protein expression levels quantitatively by Western blot analysis in human primary colorectal adenocarcinomas, adjacent normal colorectal mucosa and liver metastases. By parallel immunohistochemical staining, comparison of the expression patterns of S100A6 and Ki-67 was made between primary lesions and liver metastases and a unique S100A6 expression pattern was found in liver metastases.

### MATERIALS AND METHODS

### Surgical specimens

Fresh human tissues (primary colorectal adenocarcinoma, adjacent normal colorectal mucosa and liver metastasis from specimens resected for carcinoma) were collected from patients undergoing surgical resection in our hospital. Adjacent normal colorectal mucosa specimens were obtained from the sites approximately

*Received 7 January 2000*
*Revised 4 May 2000*
*Accepted 8 May 2000*

*Correspondence to:* K Komatsu

5–10 cm apart from the primary tumours. Surgical specimens were immediately stored at –80°C for Western blotting (33 specimens) or fixed with 10% formalin in phosphate-buffered saline (PBS) for immunohistochemistry (58 specimens). Histopathological confirmation of surgical specimens for Western blotting was performed by pathologists.

### Preparation of tissue extracts

Frozen surgical specimens were thawed, minced with scissors, crushed in a solution consisting of 0.05 M Tris-HCl, pH 6.8, 2% (w/v) SDS, 6% (v/v) *β*-mercaptoethanol and 10% (w/v) glycerol using polytron and centrifuged at 15 000 ***g*** for 10 min. The supernatant was used immediately or stored frozen at –80°C for immunoblot analysis.

### SDS-polyacrylamide gel electrophoresis (SDS-PAGE) and Western blot analysis

SDS-PAGE was performed as described by Laemmli (1970). Protein samples were electrophoresed on 15% polyacrylamide gel under reducing conditions. The resolved proteins were electrophoretically transferred to PVDF (polyvinylidene difluoride) membrane (Matsudaira, 1987). S100A6 and S100A4 were detected using monoclonal anti-pig S100A6 antibody (mAbA6) (Sigma, St. Louis, MO, USA) and rabbit anti-mouse S100A4 antiserum (polyAbA4), respectively. Actin was detected with mouse monoclonal antibody to pan-actin (Anti-Actin: Boehringer Mannheim, Mannheim, Germany). MAbA6 cross-reacts with human, bovine, rabbit and rat S100A6 and does not react with S100A2, S100A1 and S100B. Bovine S100A1 and bovine S100B were obtained from Sigma. Anti-mouse IgG (H+L) AP conjugate or anti-rabbit IgG (Fc) AP conjugate and BCIP/NBT Color Substrate (Promega, Madison, WI, USA) were used for alkaline phosphatase detection. Protein concentration was determined by protein assay (Bio-Rad, Richmond, CA, USA). Protein expression levels were quantitatively estimated by densitometric scanning using a 1200 dpi flat-bed scanner with NIH Image 1.55f. S100A6 and S100A4 protein concentrations were normalized to actin level and expressed as densitometric ratio.

### Immunohistochemical staining

Four micrometre sections from formalin-fixed, paraffin-embedded tissues were mounted on poly-L-lysine-coated slides. They were then air-dried and deparaffinized. Immunohistochemical staining was performed according to the method reported previously (Komatsu et al, 2000). Briefly, after blocking non-specific binding sites with 2% normal horse serum, the sections were incubated with mAbA6 or monoclonal anti-Ki-67 antibody (MIB-1: Immunotech, Westbroak, ME, USA). Then they were treated with biotinylated horse anti-mouse IgG (Vector, Burlingame, CA, USA) and subsequently detected with an avidin-biotin system using 0.025% 3,3′-diaminobenzidine tetrahydrochloride as a chromogen. The sections were lightly counterstained with Mayer's haematoxylin.

### Evaluation of degree of antibody reactivity

The degree of monoclonal anti-S100A6 or anti-Ki-67 antibody reactivity with each tissue section was scored by percentage of stained adenocarcinoma cells in the section. In this study, adenocarcinoma specimens with more than 50% stained cells were defined as 'Positive'. Three persons independently judged the stained cells.

### Statistical analysis

The $\chi^2$ test and Student's *t*-test were used for statistical analyses. Correlations between S100A6 and S100A4 expressions were checked by the Pearson's correlation coefficient on a per-case basis.

### RESULTS

### Western blotting of S100A6 and S100A4 in human colorectal adenocarcinomas

We first subjected recombinant rabbit S100A6, recombinant mouse S100A4, bovine S100A1, bovine S100B and human colorectal adenocarcinoma lysate to SDS-PAGE in order to examine whether mAbA6 and poly AbA4 specifically recognize S100A6 and S100A4, respectively. As shown in Figure 1, S100A6 ran faster than S100A4 in SDS-PAGE. Both antibodies specifically recognized the corresponding antigens and did not cross-react with each other. In tumour lysates, single bands were observed at the same positions as recombinant proteins.

The expression levels of S100A6 and S100A4 proteins in paired samples of human colorectal adenocarcinoma (T) and adjacent normal colorectal mucosa (N) from a patient were compared by Western blot analysis. Figure 2A shows S100A6 (upper column), S100A4 (lower column) and actin protein expressions of paired N and T from 12 cases. In 11 of 12 and seven of 12 cases, S100A6 and S100A4 expression levels were higher in T than in N, respectively. Average S100A6 level of T was significantly higher than that of N, whereas average S100A4 level of T was not (Figure 2B).

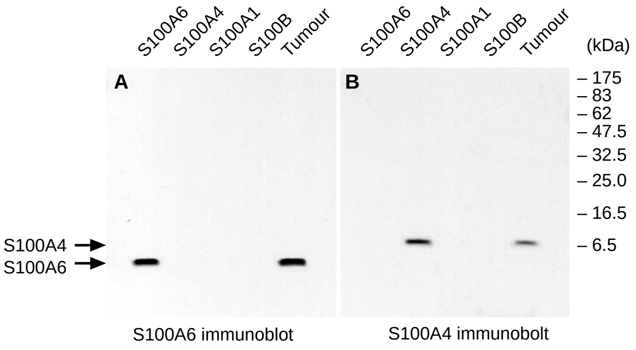

**Figure 1** Specificity of anti-S100A6 and anti-S100A4 antibodies. The recombinant rabbit S100A6 (50 ng), recombinant mouse S100A4 (50 ng), bovine S100A1 (50 ng), bovine S100B (50 ng) and human colorectal adenocarcinoma lysate (1 $\mu$g) were run on SDS-PAGE and blotted onto PVDF membrane. The membrane was cut in half and Western blotting was performed using mAbA6 (1:500) or poly AbA4 (1:1000). Arrows indicate positions of migration of S100A6 and S100A4 proteins. Positions of the molecular weight standards (in kDa) are indicated

© 2000 Cancer Research Campaign

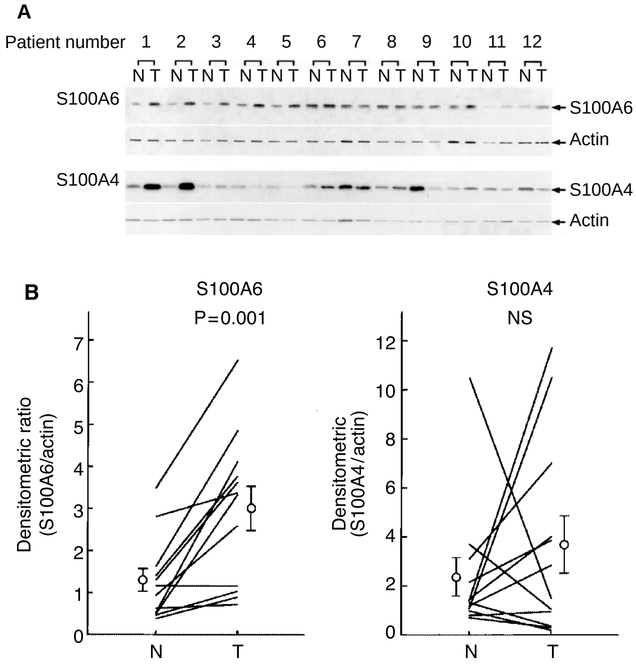

**Figure 2** S100A6 and S100A4 expressions in human primary colorectal adenocarcinomas and adjacent normal mucosa. (**A**) Western blot analyses of S100A6 and S100A4. Tissue extracts (1 $\mu$g) were run on SDS-PAGE and blotted onto PVDF membrane. S100A6 and S100A4 immunoblots in normal mucosa (N) and primary colorectal adenocarcinomas (T) in paired samples from 12 cases are shown (upper column). Actin immunoblots are also shown (lower column). Arrows indicate positions of S100A6, S100A4 and actin. (**B**) S100A6 and S100A4 expression levels in paired normal mucosa (N) and adenocarcinomas (T). Vertical bars indicate means ± SE. NS = not significant

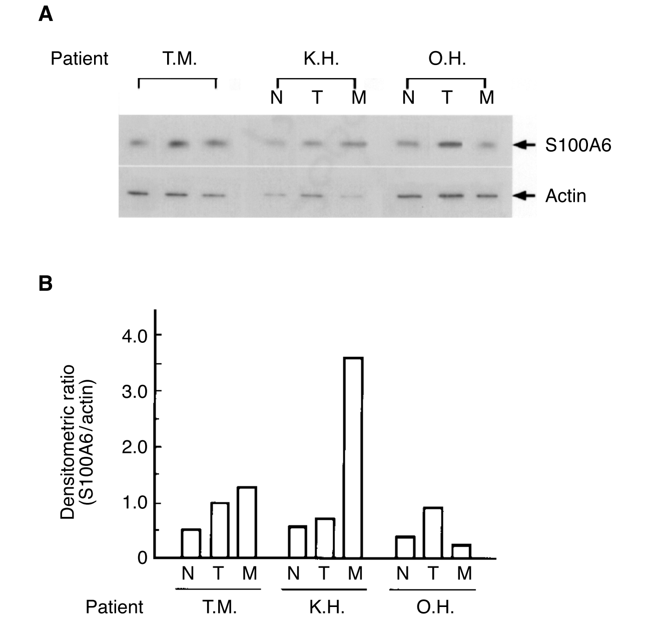

**Figure 3** S100A6 expression in human primary colorectal adenocarcinomas and liver metastases. (**A**) Western blot analysis of the S100A6. Matched primary adenocarcinoma (T), adjacent normal mucosa (N) and liver metastasis (M) tissue extracts (1 $\mu$g) from three cases were run on SDS-PAGE as described in the legend of Figure 2A. S100A6 immunoblot (upper column) and actin immunoblot (lower column) are shown. Arrows indicate positions of S100A6 and actin. (**B**) Comparison of S100A6 expression levels. The densitometric ratios (S100A6/actin) of matched N, T and M samples from three cases in Figure 3A are shown

### Expression of S100A6 in primary human colorectal adenocarcinomas and liver metastases

Three sets of matched samples, i.e. primary colorectal adenocarcinoma (T), adjacent normal mucosa (N) and liver metastasis (M) were collected from three cases and the expression of S100A6 protein was compared quantitatively by Western blot analysis (Figure 3A). As shown in Figure 3B, S100A6 expression levels were higher in M than in T in two of three cases.

### Comparison of the immunohistochemical staining patterns between S100A6 and Ki-67 in primary human colorectal adenocarcinomas and liver metastases

In order to examine the expression of S100A6 at the histological level, we performed immunohistochemical analysis using formalin-fixed paraffin-embedded surgical specimens and mAbA6. As previously reported, S100A6 was stained diffusely in whole cytoplasms in both primary colorectal adenocarcinomas and metastatic nodules in the liver (Komatsu et al, 2000). The staining was abolished when an adjacent serial section was incubated with mAbA6 that had been previously absorbed with excess recombinant rabbit S100A6 protein, and further abolished by incubating with normal mouse IgG1 (data not shown). Immunohistochemical analysis demonstrated that 23 of 42 (55%) primary colorectal adenocarcinoma and 15 of 16 (94%) metastatic nodules in the liver specimens were mAbA6-positive. We next compared the immunohistochemical staining pattern of S100A6 with that of Ki-67 in these tissues. Ki-67 is a growth marker which is present in the nuclei (especially nucleoli) of growing normal and tumour cells, although its function is still obscure (Gerdes et al, 1983). Figure 4 shows typical patterns of S100A6 and Ki-67 staining in serial sections of primary colorectal adenocarcinoma and liver metastasis. S100A6 staining was more intense in the invading fronts with structural atypia than in central portions with glandular structure of the primary adenocarcinomas (Figure 4A), while Ki-67 staining pattern was similar in these two portions (Figure 4B). Eighty five percent (35/41) of primary colorectal adenocarcinoma specimens were stained as mAbA6-positive in the invading fronts, whereas 27% (11/41) of specimens were stained as mAbA6-positive in central portions of the carcinomas (Figure 5). Only 12% (5/41) specimens had positive staining in both central portions and invading fronts. On the other hand, the percentages of Ki-67-positive adenocarcinoma specimens were similar in both portions (Figure 5).

In liver metastases, S100A6 staining was also more intense in the invading fronts than in central portions (Figure 4C), while Ki-67 staining pattern did not show such a tendency (Figure 4D). Ninety four percent (15/16) of liver metastasis specimens were stained as mAbA6-positive in the invading fronts, whereas 19% (3/16) of liver metastasis specimens were stained as mAbA6-positive in central portions (Figure 5). Only 13% (2/16) had positive staining in both central portions and invading fronts. On the other hand, Ki-67 staining pattern in the invading fronts of liver metastasis was similar to that in central portions (Figures 4D and 5).

The differences between central portions and invading fronts were statistically significant in both primary and metastatic lesions of colorectal adenocarcinomas ($P < 0.0001$).

*© 2000 Cancer Research Campaign*

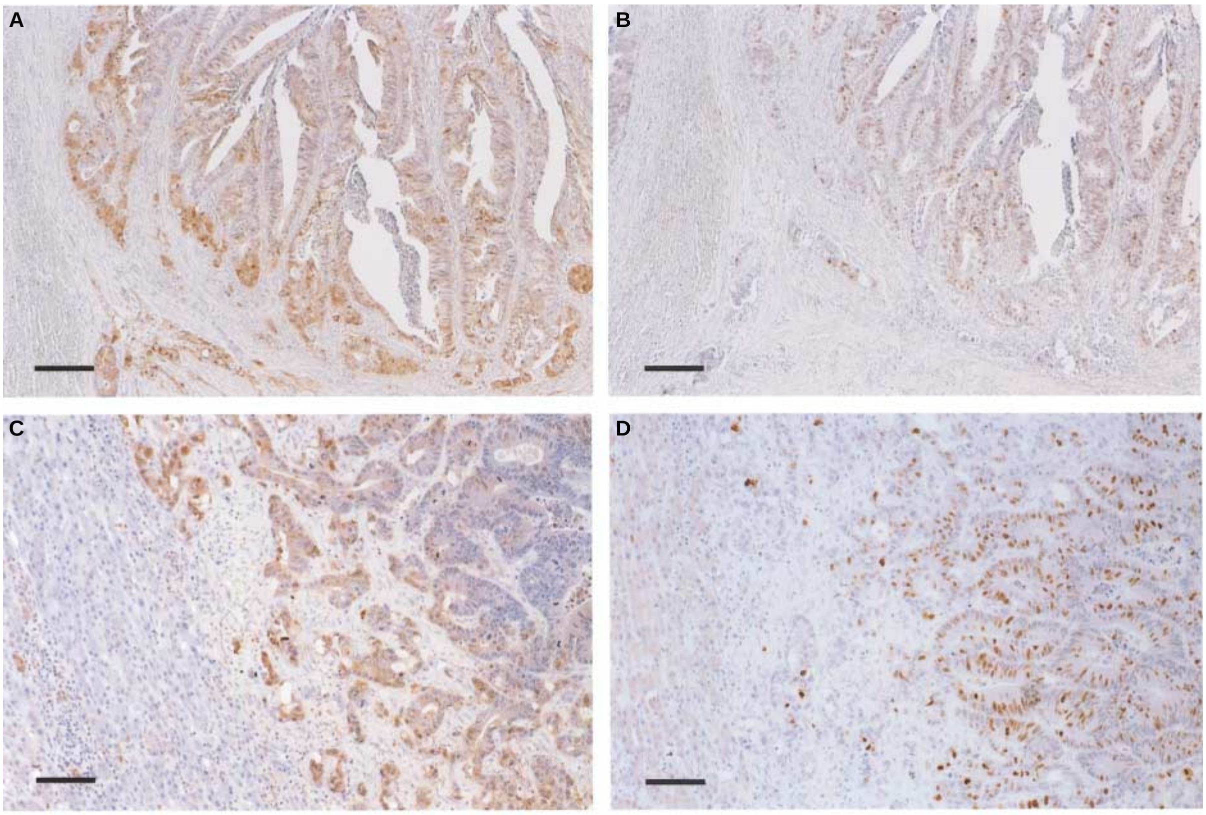

**Figure 4** Immunohistochemical staining patterns of S100A6 and Ki-67 in human primary colorectal adenocarcinoma and liver metastasis. Staining was performed as described in the text. (**A**) Primary adenocarcinoma stained with mAbA6. (**B**) Primary adenocarcinoma stained with anti-Ki-67 antibody. (**C**) Liver metastasis stained with mAbA6. (**D**) Liver metastasis stained with anti-Ki-67 antibody. A and B or C and D are adjacent serial sections. Magnification × 20. Scale bars = 100 $\mu$m.

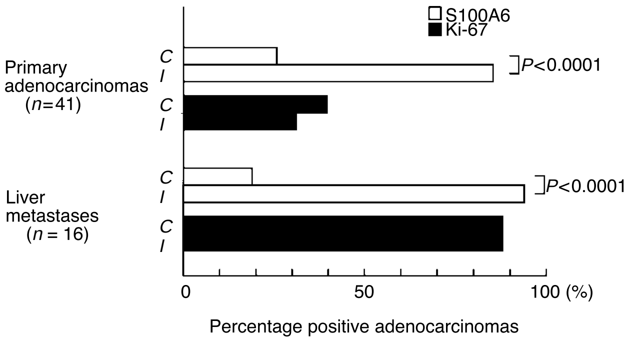

**Figure 5** Percentages of S100A6 and Ki-67 immunopositive primary and liver metastatic human colorectal adenocarcinomas. *C* = central portions of tumours. *I* = Invading fronts of tumours. Percentage positive adenocarcinomas = % of adenocarcinoma specimens with more than 50% stained cells

### DISCUSSION

Histopathological confirmation of surgical specimens for Western blotting revealed that primary colorectal adenocarcinoma specimens consisted of adenocarcinoma cells (60–70%), stromal cells (20–30%) and normal mucosal tissues (5–10%) and that liver metastasis specimens were made up of adenocarcinoma cells (70–80%) and stromal cells (20–30%). Almost no normal liver cells were observed in liver metastasis specimens. Normal mucosa specimens consisted of only non-tumorous mucosal tissues.

By Western blot analysis, we found that the expression level of S100A6 protein was significantly higher (about × 2.3) in human colorectal adenocarcinomas (T) than in paired adjacent normal mucosa (N) (Figures 2A and 2B). Although average S100A4 level in T was 1.6 times higher than in N, this difference was not statistically significant (Figure 2B). If case 9 was excluded from the samples, average S100A4 level in T was 2.4 times higher than in N. However, this difference still remained not significant. In contrast, Takenaga et al (1997) found that S100A4 mRNA expression is significantly higher in human colorectal adenocarcinomas than in normal mucosa. Because a considerable variation in S100A4 expression levels was observed in patients with primary colorectal adenocarcinoma (Figure 2A), this discrepancy may be due to variations in characteristics of patients. Concerning the expression levels of S100A6 and S100A4 in cancer tissues, Maelandsmo et al (1997) showed that S100A6 mRNA expression levels were in general higher in human metastatic melanomas than in the benign naevi, whereas there were no clear differences in S100A4 expression levels between these two tissues. Although cancer tissue is different, their results are similar to ours.

When the correlation between expressions of S100A6/actin and S100A4/actin in normal mucosa and in adenocarcinoma samples was analysed by Pearson's correlation coefficient tests on a per-case basis, no appreciable correlation could be found in both samples (data not shown). Although these two genes are known to

 © 2000 Cancer Research Campaign colocalize on the chromosome 1q21 (Schäfer et al, 1995), this suggests that expression of these two genes may be differentially regulated.

In three sets of matched samples of primary colorectal adenocarcinoma and the liver metastasis, S100A6 expression levels were higher in metastasis than in primary tumour in only two of three cases. This result suggests that metastatic nodules in the liver do not always consist of highly S100A6-positive carcinoma cells.

Therefore, the distribution of S100A6-positive carcinoma cells was examined in primary adenocarcinomas and liver metastases by immunohistochemical analysis. In primary adenocarcinomas, S100A6 was significantly more intensely immunostained in the invading fronts with structural atypia than in central portions with glandular structure (Figures 4A and 5), whereas Ki-67 staining pattern was similar in these two portions (Figures 4B and 5). Since S100A6 expression has been considered to be involved in cell growth, these results were unexpected. However, a dissociation between S100A6 expression and cell growth was shown in human endometrial adenocarcinoma cell lines in which phorbor esters inhibit cell growth but enhance S100A6 expression (Gong et al, 1992). We observed that deeply invaded adenocarcinoma cells consisting of single cells, and the carcinoma cells invaded into lymphatic vessels, were intensely stained with mAbA6. The infiltrating neutrophils and macrophages in the stroma were also mAbA6-positive (data not shown). Furthermore, Guo et al (1990) reported that S100A6 expression was higher in metastatic H-*ras* transformed NIH 3T3 cells than in non-metastatic cells. Weterman et al (1992) showed that S100A6 expression was elevated in highly metastatic human melanoma cell lines as compared with low metastatic ones. These results suggest that S100A6 is involved in the invasive process of human colorectal adenocarcinoma cells.

Carcinoma cells with high metastatic potentials in the primary lesion are often dissociated or dedifferentiated at the invading fronts (Ono et al, 1996). These dissociated carcinoma cells invade the surrounding tissues, metastasize to the liver and proliferate to form metastatic nodules. Carcinoma cells metastasized to the liver regain cell–cell adhesiveness, form glandular structure again and are often dissociated at the invading fronts of metastatic nodules. In this study, the dissociated carcinoma cells were more strongly mAbA6-positive as compared to the cells with glandular structure at the centre of the primary lesions (Figure 4A). Therefore, we expected that all of the carcinoma cells in metastatic nodules in the liver were strongly mAbA6-positive. To our surprise, however, we found that S100A6 was more intensely stained in the invading fronts with structural atypia than in central portions with glandular structure of the metastatic nodules (Figure 4C), whereas Ki-67 staining was similar in these two portions (Figure 4D). This difference in S100A6 staining was statistically significant (Figure 5: $P < 0.0001$). Normal liver cells were not stained at all with mAbA6. These results suggest that S100A6 expression in metastatic adenocarcinoma cells is reversible and that S100A6 expression levels are reduced when carcinoma cells regain cell–cell adhesiveness at the central portions of metastatic nodules.

It has been reported that Ki-67 expression in liver metastases is higher (De Jong et al, 1998) or lower (Saarnio et al, 1998) than in primary tumours and that Ki-67 expression levels are not significantly different between these two tissues (Thomas et al, 1998). In the present study, Ki-67 expression was significantly higher in liver metastases than in primary lesions (Figure 5: $P = 0.002$). Variations in characteristics of patients, tumour heterogeneity and immunohistochemical assessments are possible explanations for this discrepancy in the results.

The immunohistochemical staining pattern of S100A4 in human colorectal adenocarcinomas was reported by Takenaga et al (1997). They showed that most of the adenocarcinoma cells that invaded deeply were much more intensely stained than those in the mucosal region. Although staining patterns of S100A6 and S100A4 appeared similar in primary adenocarcinomas, their functional roles in the tumour remain to be elucidated. Further studies are in progress in our laboratory to provide evidence for a causative role of S100A6 in the invasive potential of human colorectal adenocarcinoma cells.

### ACKNOWLEDGEMENTS

We thank Dr K Takenaga, Chiba Cancer Center Research Institute, for supplying anti-S100A4 antibody and recombinant mouse S100A4. We are also grateful to Miss A Hotta and S Kitagaki for secretarial help in the preparation of the manuscript. This work was supported in part by a grant-in-aid from the Ministry of Health and Welfare for a New 10-Year Strategy for Cancer Control, Japan.

### REFERENCES

Calabretta B, Battini R, Kaczmarek L, de Riel JK and Baserga R (1986) Molecular cloning of the cDNA for a growth factor-inducible gene with strong homology to S-100, a calcium-binding protein. *J Biol Chem* **261**: 12628–12632

Davies BR, Davies MPA, Gibbs FEM, Barraclough R and Rudland PS (1993) Induction of the metastatic phenotype by transfection of a benign rat mammary epithelial cell line with the gene for p9Ka, a rat calcium-binding protein, but not with the oncogene EJ-ras-1. *Oncogene* **8**: 999–1008

De Jong KP, Stellema R, Karrenbeld A, Koudstaal J, Gouw ASH, Sluiter WJ, Peeters PMJG, Slooff MJH and De Vries EGE (1998) Clinical relevance of transforming growth factor α, epidermal growth factor receptor, p53, and Ki-67 in colorectal liver metastases and corresponding primary tumors. *Hepatology* **28**: 971–979

Ebralidze A, Tulchinsky E, Grigorian M, Afanasyeva A, Senin V, Revazova E and Lulcanidin E (1989) Isolation and characterization of a gene specifically expressed in different metastatic cells and whose deduced gene products has a high degree of homology to a $Ca^{2+}$-binding protein family. *Genes Dev* **3**: 1086–1093

Gerdes J, Schwab U, Lemke H and Stein H (1983) Production of a mouse monoclonal antibody reactive with a human nuclear antigen associated with cell proliferation. *Int J Cancer* **31**: 13–20

Gong Y, Alkhalaf B, Murphy LJ and Murphy LC (1992) Differential effects of phorbol esters on proliferation and calcyclin expression in human endometrial carcinoma cells. *Cell Growth Differ* **3**: 847–853

Guo X, Chambers AF, Parfett CLJ, Waterhouse P, Murphy LC, Reid RE, Graig AM, Edwards DR and Denhardt DT (1990) Identification of a serum-inducible messenger RNA (5B10) as the mouse homologue of calcyclin: Tissue distribution and expression in metastatic, ras-transformed NIH 3T3 cells. *Cell Growth Differ* **1**: 333–338

Jackson-Grusby LL, Swiergiel J and Linzer DIH (1987) A growth-related mRNA in cultured mouse cells encodes a placental calcium binding protein. *Nucleic Acids Res* **15**: 6677–6690

Komatsu K, Andoh A, Ishiguro S, Suzuki N, Hunai H, Kobune-Fujiwara Y, Kameyama M, Miyoshi J, Akedo H and Nakamura H (2000) Increased expression of S100A6 (calcyclin), a calcium-binding protein of S100 family, in human colorectal adenocarcinoma. *Clin Cancer Res* **6**: 172–177

Laemmli UK (1970) Cleavage of structural proteins during the assembly of the head of bacteriophage T4. *Nature* **227**: 680–685

Maelandsmo GM, Hovig E, Skrede M, Engebraaten O, Flørenes VA, Myklebost O, Grigorian M, Lukanidin E, Scanlon KJ and Fodstad Ø (1996) Reversal of the *in vivo* metastatic phenotype of human tumor cells by an *anti-CAPL (mts1)* ribozyme. *Cancer Res* **56**: 5490–5498

Maelandsmo GM, Flørenes VA, Mellingsaeter T, Hovig E, Kerbel RS and Fodstad Ø (1997) Differential expression patterns of *S100A2, S100A4* and *S100A6* during progression of human malignant melanoma. *Int J Cancer* **74**: 464–469

© 2000 Cancer Research Campaign 

Matsudaira P (1987) Sequence from picomole quantities of proteins electroblotted onto polyvinylidene difluoride membranes. *J Biol Chem* **262**: 10035–10038

Ono M, Sakamoto M, Ino Y, Moriya Y, Sugihara K, Muto T and Hirohashi S (1996) Cancer cell morphology at the invasive front and expression of cell adhesion-related carbohydrate in the primary lesion of patients with colorectal carcinoma with liver metastasis. *Cancer* **78**: 1179–1186

Saarnio J, Parkkila S, Parkkila A-K, Haukipuro K, Pastoreková S, Pastorek J, Kairaluoma MI and Karttunen TJ (1998) Immunohistochemical study of colorectal tumors for expression of a novel transmembrane carbonic anhydrate, MN/CAIX, with potential value as a marker of cell proliferation. *Am J Pathol* **153**: 279–285

Schäfer BW and Heizmann CW (1996) The S100 family of EF-hand calcium-binding proteins: functions and pathology. *Trends Biochem Sci* **21**: 134–140

Schäfer BW, Wicki R, Engelkamp D, Mattei M-G and Heizmann CW (1995) Isolation of a YAC clone convering a cluster of nine S100 genes on human chromosome 1q21: Rationale for a new nomenclature of the S100 calcium-binding protein family. *Genomics* **25**: 638–643

Takenaga K, Nakanishi H, Wada K, Suzuki M, Matsuzaki O, Matsuura A and Endo H (1997) Increased expression of S100A4, a matastasis-associated gene, in human colorectal adenocarcinomas. *Clin Cancer Res* **3**: 2309–2316

Thomas GV, Szigeti K, Murphy M, Draetta G, Pagano M and Loda M (1998) Down-regulation of p27 is associated with development of colorectal adenocarcinoma metastases. *Am J Pathol* **153**: 681–687

Weterman MAJ, Stoopen GM, van Muijen GNP, Kuznicki J, Ruiter DJ and Bloemers HPJ (1992) Expression of calcyclin in human melanoma cell lines correlates with metastatic behavior in nude mice. *Cancer Res* **52**: 1291–1296

Weterman MAJ, van Muijen GNP, Bloemers HPJ and Ruiter DJ (1993) Expression of calcyclin in human melanocytic lesions. *Cancer Res* **53**: 6061–6066

Zimmer DB, Cornwall EH, Landar A and Song W (1995) The S100 protein family: history, function and expression. *Brain Res Bull* **37**: 417–429

 *© 2000 Cancer Research Campaign*
